# Immunoglobulin D Multiple Myeloma: A Rare Variant

**DOI:** 10.7759/cureus.21912

**Published:** 2022-02-04

**Authors:** Kira N MacDougall, Muhammad Rafay Khan Niazi, Maryam Rehan, Wei Xue, Meekoo Dhar

**Affiliations:** 1 Department of Hematology and Medical Oncology, Oklahoma University of Health Sciences, Oklahoma City, USA; 2 Department of Internal Medicine, Staten Island University Hospital, Staten Island, USA; 3 Department of Hematology and Medical Oncology, Staten Island University Hospital, Staten Island, USA; 4 Department of Pathology, Staten Island University Hospital, Staten Island, USA

**Keywords:** immunoglobulin d multiple myeloma, diagnosis, survival, treatment choices, multiple myeloma

## Abstract

Immunoglobulin D multiple myeloma (IgD MM) is a rare isotype of multiple myeloma (MM), comprising less than 2% of all cases. It is often associated with advanced disease at the time of diagnosis, an aggressive clinical course, and shorter overall survival (OS) than other subtypes of MM. There is an increased frequency of undetectable or small monoclonal (M-) protein levels on electrophoresis, hypercalcemia, anemia, lytic bony lesions, and renal failure. However, given the rarity of the disease, there are few cases of IgD MM described in the literature. Given the very small amount of IgD immunoglobulins, they may form very small or undetectable M spike on electrophoresis, making the diagnostic error in diagnosing this specific subgroup very easy. Treatment for MM has seen significant advancement, especially over the last decade, with the advent of medications such as proteasome inhibitors, immunomodulatory agents, and monoclonal antibodies. It is important to understand how IgD MM responds to these newer agents and why this disease continues to be associated with poor outcomes despite advancements in treatment. Small clinical studies on patients with IgD MM show better outcomes following a combination of high-dose chemotherapy (HDCT) and autologous stem cell transplant (ASCT) compared to standard chemotherapy. Given the rarity of the disease, there are no large studies done to see the effectiveness of these treatments, and most of the data are derived from small case series. We report a case of IgD kappa MM that was incidentally discovered following a traumatic bicycle accident. The patient started treatment with bortezomib and dexamethasone (Vd) as an inpatient while he was in the rehabilitation unit and was later switched to bortezomib, dexamethasone, and lenalidomide (VRd) as an outpatient. He has now completed seven cycles and successfully underwent autologous hematopoietic stem cell transplantation.

## Introduction

Multiple myeloma (MM) is characterized by the neoplastic proliferation of a single clone of plasma cells that produce a monoclonal immunoglobulin. Clinical manifestations include normocytic anemia, bone pain, elevated creatinine, fatigue, generalized weakness, hypercalcemia, and weight loss. Immunoglobulin D multiple myeloma (IgD MM) is a rare isotype of MM, comprising less than 2% of all cases [1]. The condition is more common in males and compared with other MM isotypes, IgD MM is characterized by an aggressive clinical course with worse overall survival (OS) and a high frequency of complications [1]. The incidence of hypercalcemia and amyloid light chain (AL) amyloidosis is more common in IgD MM than in other myelomas [2].

Given the rarity of IgD MM and the fact that immunofixation for immunoglobulin D (IgD) is not routinely performed, there are few reports of IgD MM identified and even fewer reported in the literature. Most of these case reports are from the 1960s and 1980s and do not include treatment with newer agents. Free light chains (FLC) are present in the serum and urine of all patients with plasma cell disorders. Automated and sensitive FLC immunoassays have become readily available, making the diagnosis of IgD MM much easier than before. Treatment for MM has shown significant advancement over the years. Patients with IgD MM have shown to have better outcomes when treated with high-dose chemotherapy (HDCT) in combination with autologous stem cell transplant (ASCT) as compared to standard-dose chemotherapy. A study on 25 patients with IgD MM showed that ASCT had better outcomes in these patients as compared to standard-dose chemotherapy [3].

Treatment for MM is rapidly evolving. It is important to understand how IgD MM responds to these newer agents and whether this disease continues to be associated with poor outcomes despite advancements in treatment. We report a case of IgD kappa MM that was incidentally discovered following a traumatic bicycle accident.

## Case presentation

We report the case of a 64-year-old male with a past medical history of pituitary adenoma and stage 1 non-small cell lung cancer for which he underwent video-assisted thoracoscopic surgery. He presented to the emergency department following a collision with a motor vehicle while riding his bicycle. On arrival, the patient had a Glasgow Coma Scale score of 7 and was intubated for airway protection. He was found to have extensive facial fractures, sinus fractures, bilateral nasal fractures, pneumocephalus, and left frontal, temporal, and parietal subarachnoid hemorrhage. On the fourth day of admission, he underwent a closed reduction of his nasal fractures and was extubated after 10 days of hospital admission. As a result of his traumatic brain injury, the patient was left with severe cognitive deficits.

**Figure 1:**
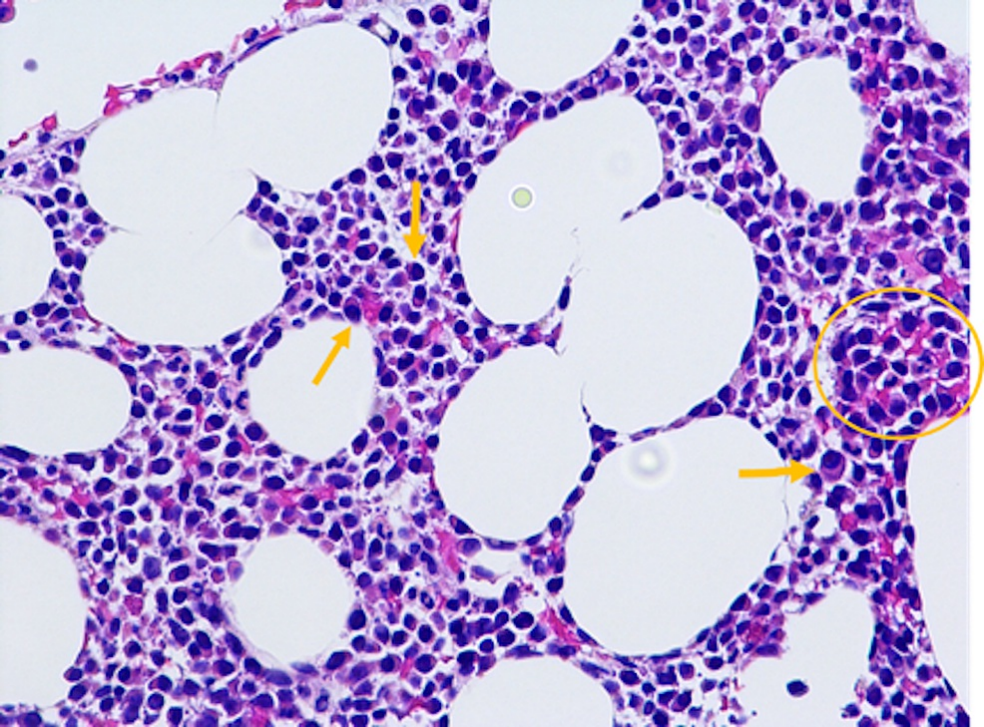
Hematoxylin and eosin-stained bone marrow biopsy demonstrating an increased number of plasma cells with mostly interstitial distribution (yellow arrows). In some areas, plasma cells forming sheet/cluster (yellow circle) can also be seen.

**Figure 2:**
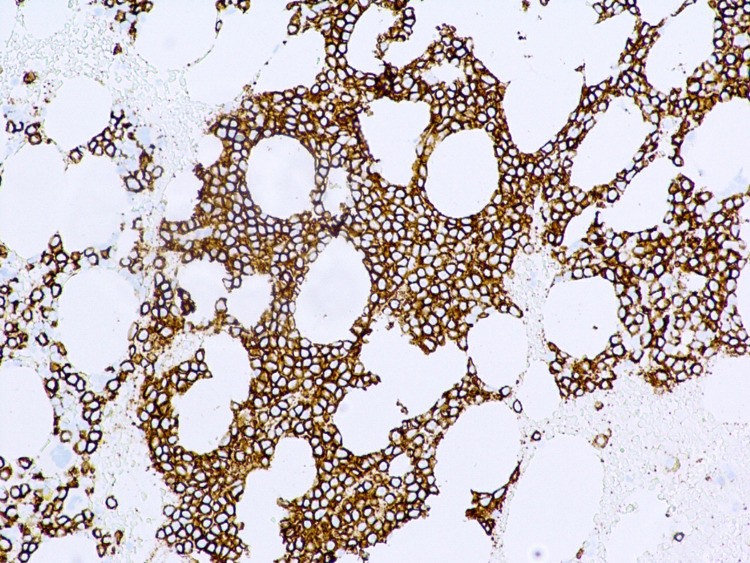
CD138 immunohistochemistry staining showing up to 90% interstitial infiltration of bone marrow by plasma cells.

Later in his hospital course, the hematology team was consulted for persistent normocytic anemia (Table 1). Serum protein electrophoresis identified three gamma-migrating paraproteins. M-1 spike was 0.2 g/dL (IgD kappa), and M-2 and M-3 spike was 0.1 g/dL (free kappa). Bence-Jones excretion in the urine was elevated at 2423.20 mg/24 hours. Workup showed a beta-2 microglobulin of 4.7 mg/L and normal lactate dehydrogenase (LDH) and albumin. The patient underwent a bone marrow biopsy, which showed an increased number of plasma cells (Figure 1). Immunohistochemistry was positive for CD138, with 90% interstitial infiltration of the bone marrow by plasma cells (Figure 2).

**Table 1:** Laboratory results. MCH: mean cell hemoglobin; MCHC: mean cell hemoglobin concentration; MCV: mean cell volume; FKLC: free kappa light chains; FLLC: free lambda light chains; LDH: lactate dehydrogenase

Parameter, unit	At diagnosis	After six cycles	Reference interval
Red blood cells, ×10^12^/L	3.00	5.98	4.70–6.10
Hemoglobin, g/L	9.0	12.9	14.0–18.0
Hematocrit, L/L	28.0	40.4	42.0–52.0
MCV, fL	93.3	87.1	80.0–94.0
MCH, pg	30.0	27.8	27.0–31.0
MCHC, g/L	32.1	31.9	32.0–37.0
Platelets, ×10^9^/L	132	213	130–400
Neutrophils, ×10^9^/L	3.52	2.79	1.40–6.50
Lymphocytes, ×10^9^/L	1.71	1.23	1.20–3.40
Monocytes, ×10^9^/L	1.22	1.49	0.10–0.60
Eosinophils, ×10^9^/L	0.10	0.12	0.00–0.70
Basophils, ×10^9^/L	0.05	0.04	0.00–0.20
Albumin, g/dL	3.5	3.9	3.6–5.5
Alpha 1, g/dL	0.3	0.3	0.1–0.4
Alpha 2, g/dL	0.6	0.7	0.5–1.0
Beta globulin, g/dL	0.6	0.7	0.5–1.0
Gamma globulin, g/dL	0.6	0.6	0.6–1.6
M spike			
M-1 spike (IgD kappa), g/dL	0.2	Unable to quantify	0.0–0.0
M-2 and M-3 spike (free kappa), g/dL	0.1	Unable to quantify	0.0–0.0
Immunoglobulin G, mg/dL	501	515	610–1660
Immunoglobulin A, mg/dL	71	99	84–499
Immunoglobulin M, mg/dL	<10	20	35–242
Immunoglobulin D, mg/dL	351	1	<12
Immunoglobulin E, KU/L	<2	-	<100
FKLC, mg/dL	453.58	1.54	0.33–1.94
FLLC, mg/dL	0.65	0.77	0.57–2.63
FKLC/FLLC ratio	697.82	2	0.26–1.65
Creatinine, mg/dL	0.7	0.9	0.7–1.5
Urea, mg/dL	10	16	10–20
Total protein, g/dL	5.6	6.2	6.0–8.3
Calcium, mg/dL	8.8	9.3	8.5–10.1
Beta-2 microglobulin, mg/L	4.7	2.1	0.8–2.2
LDH, U/L	217	205	50–242

Full-body CT scans did not show any lytic lesions. He was found to have standard-risk cytogenetics such as t(11;14) and was negative for high-risk cytogenetics such as t(14;16), t(14;20) del17p13, gain 1q, and/or del1p32. He was diagnosed with Revised Multiple Myeloma International Staging System (R-ISS) Stage II IgD kappa MM. As an inpatient, he completed one cycle of bortezomib and dexamethasone (Vd).

After several months of intensive inpatient physical rehabilitation, he was discharged home. He then completed seven cycles of bortezomib, lenalidomide, and dexamethasone (VRd). After six cycles, the patient’s kappa light chains decreased from 453 mg/dL to 1.54 mg/dL, and the patient achieved a complete response according to the International Myeloma Working Group (IMWG) classification. The patient successfully underwent autologous hematopoietic stem cell transplantation without complications. He is now on maintenance therapy with lenalidomide. He is monitored with regular complete blood count (CBC), complete metabolic profile (CMP), LDH, serum protein electrophoresis (SPEP) with immunoelectrophoresis (IFE), free light chains, and IgD and continues physical and occupational therapy for his traumatic brain injury.

## Discussion

IgD was first discovered as a unique myeloma immunoglobulin in 1965. Since then, studies have estimated a prevalence of IgD MM of less than 2% of all myeloma cases. However, this may underestimate the true prevalence of the disease because immunofixation for IgD is not routinely performed and the level of IgD in the serum is extremely low (1-10 mg/dL), which may cause a small or unrecognizable M protein spike on electrophoresis. Therefore, many cases of IgD MM may go undetected or given a false diagnosis of nonsecretory or light chain myeloma.

Although the clinical features of IgD MM are similar to those of other myeloma types, IgD MM is characterized by a more aggressive clinical course with a high frequency of renal failure, hypercalcemia, amyloidosis, extramedullary plasmacytomas (EMP), extraosseous lesions, hepatosplenomegaly, generalized lymphadenopathy, and a poor prognosis [1,4]. A 1975 study of 133 patients with IgD MM reported a median survival of only 13.7 months, while another study in 1994 of 53 patients with IgD MM reported a median survival of 21 months [1,4]. Despite these studies, two case reports were identified in the literature of patients with IgD MM achieving complete remission at eight and 21 years follow-up [5], indicating that some patients do very well. A case series of 53 patients in 2012 showed that IgD MM is associated with a lower incidence of renal amyloidosis, higher serum albumin, and lower urine protein levels [2]. Where other IgM and IgA myelomas have a high concentration of serum proteins and M components, patients with IgD MM have low M component proteins, which can be undetectable in some cases. There is a remarkable preponderance of λ-type light chains in these patients as compared to only one-third concentration in IgA and IgG myelomas. Patients with IgD MM have higher serum-free Bence-Jones proteins. Since free Bence-Jones proteins are not picked up on electrophoresis or buried under IgD M component spike, their incidence is underreported. Thus, the true incidence of Bence-Jones proteinemia may be higher in IgD MM than in other types of MMs [1]. Shimamoto et al. reviewed the data from 165 Japanese patients with IgD MM classified according to the Durie-Salmon (DS) staging system [6]. Another similar study comprised 379 patients with IgD MM. However, both studies failed to prove any correlation between the DS stage and OS [6]. In the end, Shimamoto et al. deduced that white blood cell count (WBC) < 7 × 10^9^/L versus > 7 × 10^9^/L light chain subtype (lambda versus kappa) were important predictors of survival, showing better OS associated with a lower WBC count and kappa light chains.

Amyloidosis has been reported to be common among patients with IgD MM. In an autopsy series of 23 patients, 10 patients (44%) were found to have amyloidosis [7]. In another case series of 53 patients, amyloidosis, carpal tunnel syndrome, macroglossia, peripheral edema, fatigue, peripheral neuropathy, and hepatic, renal, cardiac involvement were reported as presenting complaints. These patients were compared with 144 cases of non-IgD monoclonal proteins, and cardiac amyloidosis was found in 45% of IgD cases as compared to 56% of non-IgD patients (P = 0.047). Renal amyloidosis was found in 36% versus 58% of these two groups, respectively (P = 0.05). Survival outcomes were found not to be different among IgD amyloidosis as compared to IgG, IgA, or light chain myeloma amyloidosis [2].

In advanced disease, plasma cells become independent of the bone marrow microenvironment, and they spread to peripheral blood, manifesting as soft tissue plasmacytoma or plasma cell leukemia (PCL), which is defined as peripheral blood plasma cells > 2 × 10^9^/L and/or >20% plasma cells in the peripheral blood [8]. PCLs are usually common among the elderly and associated with poor outcomes. A higher incidence of t(11;14) (q13;q32) is reported to be associated with PCL [9]. IgD MM can present with hepatomegaly or splenomegaly [1]. Other presentations include a high rate of extramedullary plasmacytoma (EMP), which may present as nerve root compression or extradural tumor. Studies have shown that patients with EMP have decreased progression-free survival (PFS) (18 months versus 30 months; P = 0.03), but there are no significant changes in overall survival (OS) (36 months versus 43 months; P = 0.36) [10]. Hobbs et al. described these EMPS as 1) tumors breaking the cortex of the bones and growing locally or (2) those developing within soft tissues [11]. Bladé et al. reported EMP incidence in 10 of 53 patients (19%) with IgD myeloma [4]. Eight additional patients developed EMP later during the course of their disease, and seven out of the 10 patients had extradural tumors [4].

Liu et al. studied the characteristics of 365 patients with IgD myeloma compared with non-IgD myeloma patients [12]. The frequency of t(11;14) was found to be predominantly higher in patients with IgD myeloma as compared with non-IgD myeloma patients (P < 0.001) [12]. One of the clinical correlations for t(11;14) in these patients can be the increased expression of BCL-2 proteins. These cytogenetic abnormalities were not found to have any impact on OS. Venetoclax may be used to inhibit this anti-apoptosis protein for the treatment of IgD myeloma in these patients. Other chromosomal abnormalities most frequently coupled with t(11;14) were 13q, 1q21+, followed by 17p translocation. Double-hit and triple-hit translocation occurred in 5.5% and 0.3%, respectively, of the patients only [12]. These cytogenetic aberrations are not shown to have any prognostic effect on OS [12].

Although the overall survival (OS) of patients with IgD MM is shorter than patients with other MM subtypes, the outcome for patients with IgD MM is improving with the use of novel agents and autologous stem cell transplantation [13]. Drugs such as bortezomib, a proteasome inhibitor, and lenalidomide, an immunomodulatory agent, have proven to be effective against the disease [14], along with autologous hematopoietic stem cell transplantation (ASCT). The anti-CD38 monoclonal antibody daratumumab is now approved by the US Food and Drug Administration (FDA) for patients with newly diagnosed or relapsed and refractory MM, in various combinations with dexamethasone, a proteasome inhibitor and an immunomodulatory drug [15]. Due to similar efficacy, an improved safety profile, and decreased cost, it is now recommended to administer subcutaneous daratumumab in combination with hyaluronidase rather than intravenous daratumumab [16]. Even more recently, belantamab mafodotin, an anti-B-cell maturation antigen (BCMA) humanized immunoglobulin G antibody conjugated to the microtubular disrupting agent monomethyl auristatin, has been approved as a single agent by the FDA for patients who have received at least four prior therapies, including an anti-CD38 monoclonal antibody, a proteasome inhibitor, and an immunomodulatory agent. Ciltacabtagene autoleucel, a chimeric antigen receptor T-cell therapy against BCMA, demonstrated durable responses in heavily pretreated patients with MM and was granted a breakthrough therapy designation by the FDA [17]. This adds to the growing list of novel agents to treat MM and highlights promising areas of future research.

The assessment of patients with suspected IgD myeloma starts with a comprehensive history and physical examination. As mentioned before, IgD myeloma is a diagnostic challenge since the serum concentration of IgD immunoglobulin can be very low, and it can easily escape detection via electrophoresis. All patients with myeloma with free serum light chain and no IgD or IgA M protein must be screened for the presence of IgD and IgE. These patients might be diagnosed with nonsecretory or light chain myeloma, and IgD myeloma can be overlooked initially.

In addition to these advances in therapy, ASCT is considered a cornerstone of MM therapy. Studies have shown improved outcomes in patients with IgD MM treated with high-dose chemotherapy followed by ASCT compared to chemotherapy alone. One study by Maisnar et al. reported 10 patients with IgD MM treated with high-dose chemotherapy (HDCT) followed by ASCT and 13 patients treated with conventional chemotherapy alone [18]. The median progression-free survival (PFS) was 18 months for patients treated with HDCT and 20 months for patients treated with chemotherapy alone [18]. The median OS was only 16 months for patients in the chemotherapy alone group, while the median OS for the HDCT followed by ASCT group had not yet been reached by the time the study was published [18]. The authors concluded that the overall response rate for patients with IgD MM aged 65 or less treated with HDCT and ASCT was like that seen in other MM subtypes. Another study by Pisani et al. reported six patients with IgD MM who received HDCT and 11 patients who underwent HDCT with ASCT [19]. In the group treated with HDCT, the OS was 34 months, and the PFS was 18 months [19]. OS and PFS were not reached at the time the analysis was performed. The authors concluded that the use of HDCT combined with ASCT appears to improve the prognosis of IgD MM. A study comprising 365 patients with IgD myeloma by the Asian Myeloma Network (AMN) showed that patients who received immunomodulators (thalidomide) had relatively longer OS. Patients who received ASCT had a median OS of 45.7 months, slightly longer than 35 months for non-ASCT patients [12]. In another study, where melphalan was used in patients with IgD MM, poor outcomes were noted in these patients as compared with patients with other MM following ASCT [20]. New treatment strategies, including new drugs before or after ASCT to induce a high-quality response, may improve patient outcomes in patients with IgD MM.

## Conclusions

IgD MM is a rare isotype of MM and is likely underrepresented in the literature. To accurately make the diagnosis, all patients with an elevated monoclonal light chain and negative IgG or IgA M protein must be screened for IgD presence. Historically, IgD MM has been associated with advanced disease at the time of diagnosis, an aggressive clinical course, and shorter OS compared to other subtypes of MM. However, this data was derived from before agents such as bortezomib, lenalidomide, daratumumab, and belantamab mafodotin, and ASCT were available. The treatment of MM continues to evolve, and new treatment strategies are being developed for these patients. Further research in IgD MM is needed to help us better understand the biology of this rare disease and improve patient outcomes.
